# Effects of exercise on muscle strength and characteristics in rheumatic diseases and sarcopenia: Protocol for the Care for Muscle (C4M) Study

**DOI:** 10.1371/journal.pone.0356181

**Published:** 2026-09-09

**Authors:** Maia M. Sobejana, Rosa M. Korpershoek, Maarten M. Steinz, Conny J. van der Laken, Marike van der Leeden, Richard T. Jaspers, Bart Visser, Martin van der Esch, Carel G. M. Meskers, Mariëtte de Rooij

**Affiliations:** 1 Amsterdam University Medical Center, Department of Rehabilitation Medicine, Location VU University Medical Center, Amsterdam, The Netherlands; 2 Reade, Center for Rehabilitation and Rheumatology, Amsterdam, The Netherlands; 3 Amsterdam University Medical Center, Department of Rheumatology and Clinical Immunology, Location VU Medical Center, Amsterdam, The Netherlands; 4 Department of Human Movement Sciences, Faculty of Behavioural and Movement Sciences, Amsterdam Movement Sciences, Vrije Universiteit Amsterdam, Amsterdam, the Netherlands; 5 Vrije Universiteit Amsterdam, Laboratory for Myology, Department of Human Movement Sciences, Faculty of Behavioral and Movement Sciences, Amsterdam Movement Sciences, Amsterdam, The Netherlands; 6 Amsterdam University of Applied Sciences, Center of Expertise Urban Vitality, Faculty of Health, Sport and Physical Activity, Amsterdam, The Netherlands; University of Brasilia, BRAZIL

## Abstract

**Objective:**

Muscle weakness is prevalent in rheumatoid arthritis (RA), osteoarthritis (OA) and sarcopenia (SARC). Endurance exercises may improve mitochondrial function and oxidative capacity, while strength exercises are thought to stimulate myofibrillar protein synthesis. This study aims to compare the effects of strength and endurance exercise and explore the association between muscle characteristics and exercise outcomes in patients with RA, OA and SARC. We hypothesize that responses to endurance and strength exercises in patients with muscle weakness are influenced by intramuscular pathology including muscle morphology, mitochondrial function, and systemic inflammation, based on their disease pathology, potentially requiring personalized training schedules.

**Methods:**

This two-arm, parallel-group exploratory trial will enroll 69 patients (23 RA, 23 OA, 23 SARC), randomized to endurance (n = 35) or muscle strength exercises (n = 34), using minimization to balance disease type and gender. The 8-week intervention includes two supervised sessions per week using a controlled cable pulley device (Reforter™) and fitness equipment, plus one weekly home-based session. The primary outcome is isokinetic muscle strength (peak torque), measured with the Biodex system**®**. Secondary outcomes include muscle morphology, mitochondrial function, systemic inflammation and muscle endurance (by Biodex and 6 Minute Walk test). Muscle morphology will be assessed via 3D ultrasound imaging of the vastus lateralis. Mitochondrial function will be analyzed using high-resolution respirometry on muscle biopsies. Systemic inflammation will be measured using multiplex assays or ELISA on serum samples.

**Discussion:**

This study will explore differential responses to muscle endurance and muscle strength exercises in patients with RA, OA, and SARC, offering novel insights into the molecular mechanisms of muscle weakness. The findings may help identify potential mechanisms underlying variability in exercise response and provide effect size estimates to guide future confirmatory studies and more targeted exercise interventions.

**Trial registration:**

ClinicalTrials.gov NCT06480643 (date of registration28-06-24).

## Background

The number of people aged 60 years and older worldwide is expected to double over the next 30 years, with a triple- rise in those aged over 80, leading to a dramatic rise in the prevalence of age-related diseases such as (osteo)arthritis and sarcopenia (SARC, age-related low muscle quantity and quality) [1,2]. Individuals with rheumatoid arthritis (RA) and osteoarthritis (OA) experience a 25–50% reduction in muscle strength even despite treatment induced remission of arthritis in RA [3–5], while in sarcopenia, muscle strength declines by an estimated 10–40% [6]. Low muscle quantity and quality, defined as muscle mass, strength and performance, are associated with detrimental clinical outcomes with a high socioeconomic burden as loss of independence, increased fatigue and (co)morbidity [7–9] including falls and fractures [10,11]. Exercise training can counteract low muscle quantity and quality [11,12]. In addition, to improvements in muscle strength and physical performance, exercise induces multiple biological adaptations, including improved mitochondrial function and enhanced oxidative capacity, modulation of inflammatory pathways, and improved cellular stress responses such as reduced endoplasmic reticulum stress [13]. According to current evidence, exercise should be delivered frequently [14,15]. Although group-level comparisons of exercise modalities often report similar clinical outcomes [15], inter-individual variability suggests that muscle-specific characteristics may mediate differential responses to exercise [16].

In RA, muscle weakness cannot be attributed to a decrease in muscle mass alone but is most likely also linked to impaired mitochondrial functioning mediated by chronic systemic inflammation [3,17–19]. While muscle weakness is known to be present in OA patients, the underlying causes and the pathophysiology are still unclear [20]. In SARC, muscle weakness is associated with increased muscle wasting [21,22] and decreased sensitivity to protein synthesis (anabolic resistance) [23]. Endurance exercises, generally conducted at lower intensities (30% to 50% of 1RM) and performed to volitional failure, has been shown to improve local muscular endurance and stimulate mitochondrial adaptations, thereby increasing oxidative capacity within skeletal muscle [24–26]. In contrast, strength exercises, typically performed at intensities ranging from 60% to 90% of one-repetition maximum (1RM), [24] has been shown to promote myofibrillar protein synthesis [27–30]. The optimal loading strategy for combating muscle weakness in diseases with distinct muscle pathologies such as RA, OA, and SARC remains to be investigated.

Although RA, OA and SARC represent distinct clinical entities, they all present with reduced muscle strength and impaired physical functioning. The mechanisms underlying muscle weakness are, however, expected to differ between these conditions, ranging from chronic systemic inflammation in RA, to local joint-related and low-grade inflammatory processes in OA, and age-related muscle degeneration in sarcopenia [12]. These differences in underlying muscle characteristics may contribute to the substantial inter-individual variability observed in response to exercise and highlight the need for a better understanding of mechanism-based exercise approaches [16]. Therefore, this study aims to compare the effects of strength and endurance exercise and explore the association between muscle characteristics and exercise outcomes in patients with RA, OA and SARC. We hypothesize that responses to endurance and strength exercises in patients with muscle weakness are influenced by intramuscular pathology including muscle morphology, mitochondrial function, and systemic inflammation.

## Methods

### Study design

**Fig 1:**
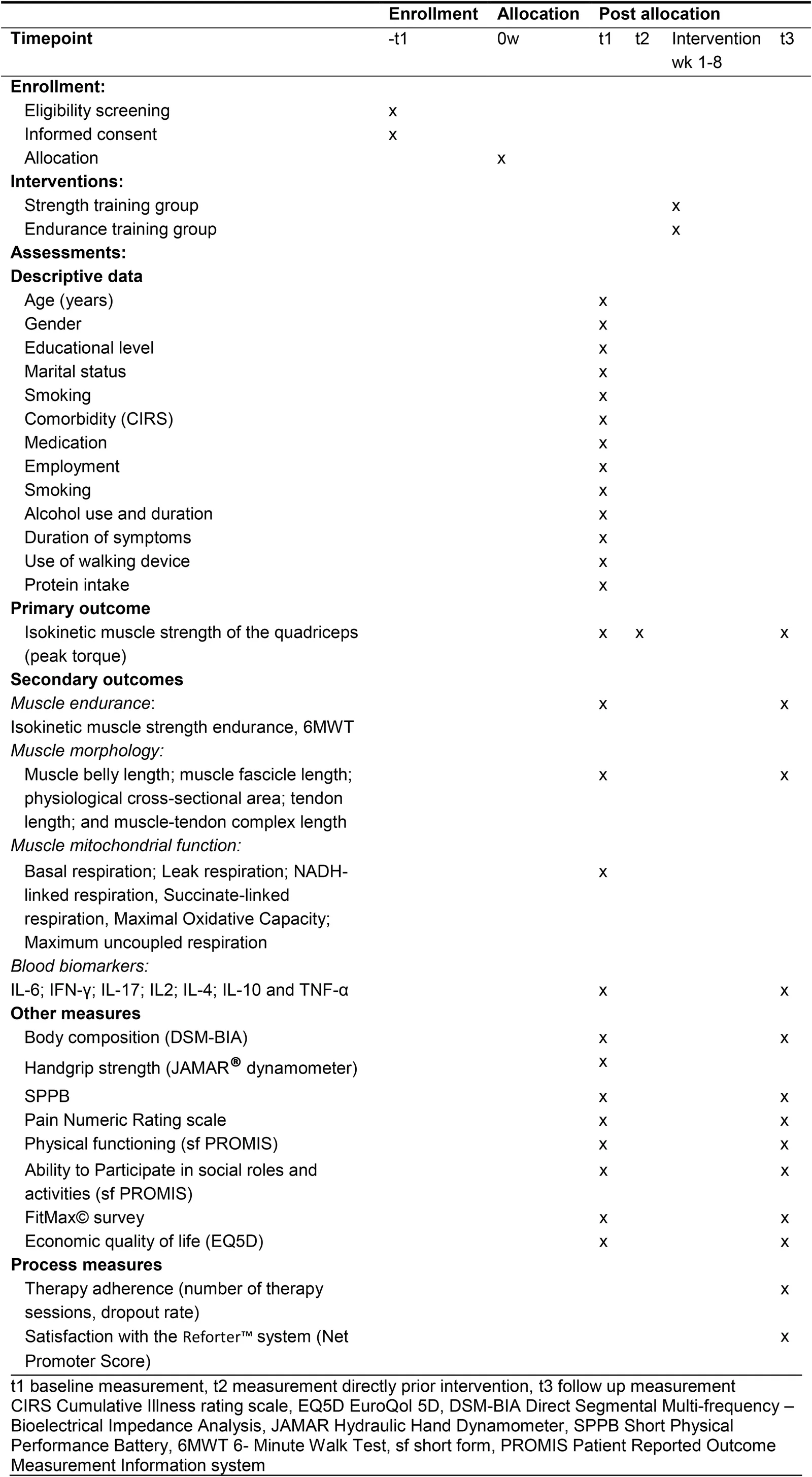
Schedule of enrollment, intervention and assessments (SPIRIT).

This study is a two-arm parallel-group exploratory trial. Participants will be randomized using a minimization procedure for allocation to either the muscle endurance or muscle strength exercise group (see section Randomization, blinding and allocation concealment). Assessments will be conducted at baseline and after 8 weeks (Fig 1) of intervention (post-treatment). Exercise training will be delivered at the outpatient clinic at Reade, center for rehabilitation and rheumatology in Amsterdam, as part of regular clinical care.

The trial is designed according to the SPIRIT (Standard Protocol Items: Recommendations for Interventional Trials) guidelines [31] and principles of Good Clinical practices.

### Ethics approval and consent to participate

Ethical approval was gained from the University Medical Center Amsterdam Research Ethics Committee (ID study NL86908.018.24). All participants must provide consent to participate. In a telephone call investigators answer any questions about study and/or consent form. Consent is obtained via a paper form. The study will be conducted in accordance with the declaration of Helsinki principles.

### Trial registration

The study is registered at ClinicalTrials.gov NCT06480643.

### Declaration of generative AI and AI-assisted technologies in the writing process

During the preparation of this work, the authors used ChatGPT- 5.5 to assist with grammar and language editing. After using this tool, the authors reviewed and revised the text as necessary and take full responsibility for the final content of the publication.

### Participants

A total of 69 participants, aged 50–80 years, will be recruited for the study: 23 participants diagnosed with RA, 23 with OA, and 23 with SARC. The inclusion and exclusion criteria are presented in Table 1. Participants will be recruited through multiple channels, e.g., referral by rheumatologists, rehabilitation physicians.

**Table 1:** Inclusion and exclusion criteria.

Inclusion Criteria
**For all patients:**
Age 50–80 years
Willingness and motivation to exercise
Low muscle strength represented by hand grip strength (HGS) <27 kg (males) and <16 kg (females). If HGS is not possible due to pain or joint deformity, use chair stand test (>15s or inability without arms) [56]
Gait speed >0.8 m/s to exclude severely disabled patients [56]
**RA patients:**
Diagnosed with RA according to EULAR/ACR criteria [57]
Disease activity score (DAS28) 2.6–5.6 (EULAR criteria), de novo or despite DMARD therapy
Stable disease (no flare/change in medication) ≥3 months
No corticosteroid use in the last 3 months
Disease duration >1 and <15 years
**OA patients:**
Knee and/or hip OA according to ACR criteria [58]
Kellgren & Lawrence grade 2–4 for hip/knee OA [42]
CRP < 10 mg/L within 3 months prior to enrollment [59]
**Sarcopenia patients:**
Sarcopenia without arthritis or other joint diseases according to the EWGSOPII criteria [56] of low muscle strength defined as HGS < 27 kg and <16 kg for males and females respectively (dynapenia). This group will primarily involve participants with probable sarcopenia (dynapenia) but may also encompass participants with confirmed sarcopenia (appendicular muscle Lean Mass (ALM)/height2 < 7.0 kg/m^2^ for males and <5.5 kg/m^2^ for females) as this is not a selection criterion. Severe sarcopenia will be excluded (gait speed <0,8 m/s)
**Exclusion criteria**
BMI < 18 or >35 kg/m²
Contraindications for exercise based on the physical activity readiness questionnaire (PAR-Q) or history of severe heart conditions (e.g., heart failure, MI < 3 months, other cardiac diseases)
Use of beta-blockers during intervention
Neurological, cachectic, inflammatory diseases or co-morbidities affecting muscle quality (e.g., MS, ongoing cancer treatment, radiotherapy/chemotherapy in last 6 months, hyperthyroidism, diabetes type 1/2, long COVID)
Participation in regular high-load training (>2x/week) within 2 months before enrollment
Recent ligament/muscle tears or injuries (<6 months)
Use of performance-enhancing drugs or supplements affecting muscle mass (e.g., protein powder)
Inability to schedule exercise therapy
Insufficient Dutch language skills or no informed consent

ACR American College of Rheumatology, CRP C-reactive protein, DMARD Disease Modifying Anti-Rheumatic Drug, EULAR European League Against Rheumatism, MS multiple sclerosis, OA Osteoarthritis, PARQ Physical Activity Readiness Questionnaire, RA Rheumatoid arthritis, SARC Sarcopenia.

### Procedures overview

**Fig 2:**
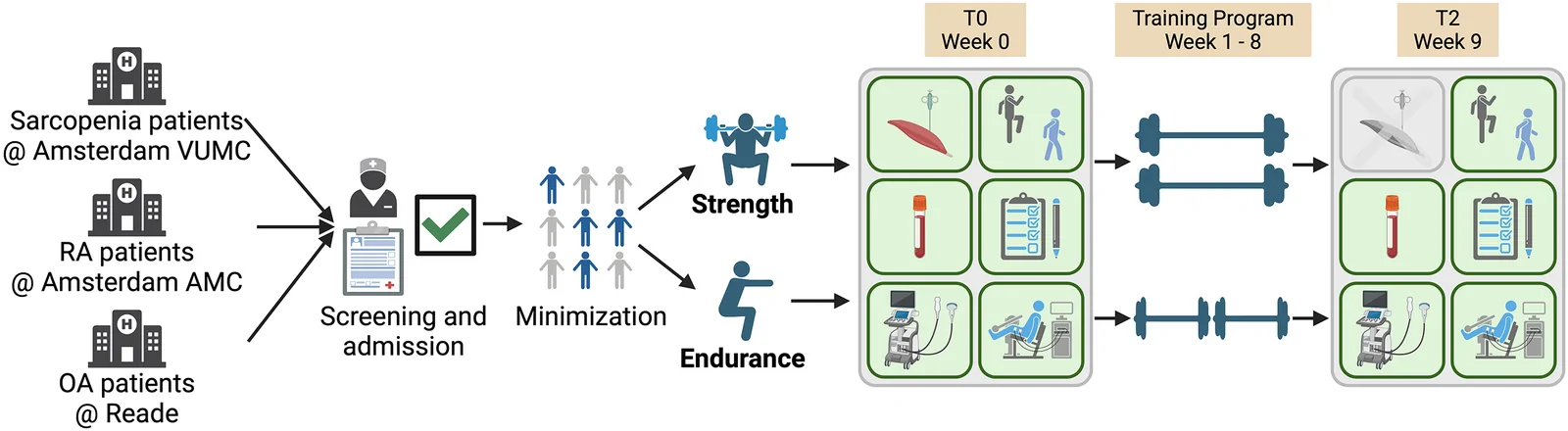
Overview trial logistics: Participants with RA, OA, or SARC are recruited and screened for eligibility. After informed consent (IC), they are randomized (minimization) to the endurance or strength exercise group. Baseline assessments (T0, week 0) include biopsy, performance tests, blood, questionnaires, imaging, and isokinetic muscle strength measures. The exercise program starts within a maximum of 2 weeks after baseline assessment. After an 8-week supervised intervention, follow-up assessments (T2, week 9) repeat all baseline measures except the biopsy.

Fig 2 provides an overview of the trial logistics. RA patients will be recruited from the Department of Rheumatology at both Reade and Amsterdam University Medical Center (location AMC). Newly referred OA patients to the Department of Rehabilitation at Reade will be invited to participate. Additionally, OA patients from an existing OA cohort at Reade, who have previously consented to be contacted for future research, will be informed about the study via email. These include patients who received treatment at Reade between 2020 and 2023. SARC patients will be recruited from the Department of Rehabilitation Medicine at the Amsterdam University Medical Centre. Patients with RA, OA, or SARC, who are deemed eligible for participation by their medical doctor (rehabilitation physician or rheumatologist) and meet the study criteria will be informed about the study by their specialist. Interested patients will receive an information letter and an IC form from their physician. Subsequently, the investigator will contact these patients by phone after one week to provide a verbal explanation of the study and to review and confirm the inclusion and exclusion criteria. If the patient expresses interest and appears to meet the criteria, an appointment will be scheduled for a screening visit with the investigator. After signing the IC form, the individual will be considered a study participant. During the screening visit, participants will perform a handgrip strength test or walk test, as both tests are indicative of general muscle strength. These tests will be used to exclude participants who are either too strong or too weak to participate (see Table 1 for inclusion criteria). Participants with moderate muscle weakness are included to ensure adequate training tolerance and to allow detection of exercise-induced improvements in muscle strength (primary outcome: peak torque). Final eligibility will be assessed by the primary investigator in consultation with a clinical team, including a rehabilitation physician and/or rheumatologist. Participant recruitment is expected to commence in January 2026 and is anticipated to conclude in August 2027. The total study duration, including follow-up and data analysis, is expected to extend beyond this period.

### Randomization, blinding and allocation concealment

We will use minimization to ensure that the groups are maximally balanced with respect to the most important stratification factors, i.e., disease (RA, OA, SARC) and gender [32]. Randomization will be performed using a centralized, computer-generated minimization procedure via an open-source online tool (OXMAR; [33]). The personnel responsible for participant enrollment will not have access to the random allocation sequence. Outcome assessors and data analysts will be blinded to treatment allocation. Participants and physiotherapists will not be blinded due to the nature of the intervention.

### Physiotherapists and training

Six physiotherapists from Reade, all experienced in treating patients with rheumatic diseases, will be trained to deliver the standardized exercise program. Prior to the start of the trial, the physiotherapists will complete a mandatory, comprehensive two-hour training session. The training includes an explanation of the intervention protocol and study procedures, followed by a one-hour practical session to familiarize the physiotherapists with the use of the training equipment and performing an adequate 10 repetition maximum (10 RM) test. Interim training sessions will be held during the trial to control and optimize the quality of the interventions.

### Intervention

The intervention consists of an 8-week exercise program. Participants will be randomized into the endurance or strength exercise group. Each week, participants complete three exercise sessions: two supervised by PTs in groups of max. four participants and one performed independently at home. Each session includes 6–8 full-body exercises from a predetermined list: three quadriceps-focused exercises, one hip or glute exercise, and two upper body exercises as appropriate (see S1 File). Exercises are performed using the Reforter™ (HapticLink, The Netherlands), fitness equipment (e.g., leg press), and resistance bands. The Reforter™ is an electronic pulley cable system that allows for: 1) concentric and eccentric muscle loading; 2) force adjustment based on position, speed, or reaction force; 3) instantaneous measurements of force [N], speed [m/s], position [m], power [W], and work [J]. The software allows for various settings and programs specific to endurance and strength focused training and provides feedback during the exercises. Physiotherapists use this data to tailor and adjust training loads.

After familiarization with the correct execution of exercises (week 1, initial three sessions), a 10-repetition maximum (RM) test will be performed for the exercises conducted on regular fitness equipment. The 10RM results are used to calculate the estimated 1RM using the Brzycki formula [34]. This process will be repeated in week 5, after which training intensity will be adjusted, and again in week 8.

Supervised sessions include a 5–10 min warm-up and a 5 min cool-down, with total session duration ranging from 45 to 60 minutes. All participants will perform the same exercises at an intensity of a of 7–8 on a 10-point physical exertion scale (see Additional file 1 supervised exercise).

The endurance group performs the exercises with a load of 30–45% of their 1RM, while the strength group performs the exercises with a load of 60–75% of their 1RM. For further details on sets, repetitions, and rest time, see Table 2.

**Table 2:** Exercise intervention of the supervised and home-based training for both endurance and strength group.

	Frequency	Intensity	Time
**Strength group**	2x/week at Reade/ supervised	10 repetitions 3 sets 90s rest between sets 60-75% 1RM	45-60mins (incl. warm-up & cool down); Progressive
	1x/week home-based/unsupervised	10 repetitions 3 sets	30 mins
**Endurance group**	2x/week at Reade/ supervised	20 repetitions 3 sets 60s rest between sets 30-45% 1RM	45-60mins (incl. warm-up & cool down); Progressive
	1x/week home-based/unsupervised	20 repetitions 3 sets	30 mins

RM: repetition maximum.

To ensure comparable workload between both groups, total training volume is calculated as [35]:


$$\begin{array}{c} Total\;training\;volume\;=\;sets*repetitions*load \end{array}$$

(see Table 3 for sample calculation). During each session, clinical parameters are monitored, and exercises will be adapted as needed.

**Table 3:** Sample workloads and total volume for endurance and strength exercises for each session, assuming 1RM = 100 kg. Total volume = sets*repetitions*load.

	Sets	Repetitions	Load	Volume	Total Volume (kg)
**Strength**					
Weeks 1–2	3	10	60% 1RM	3 sets * 10 reps * 60 kg	1800
Weeks 3–4	3	10	65% 1RM	3 sets * 10 reps * 65 kg	1950
Weeks 5–6	3	10	70% 1RM	3 sets * 10 reps * 70 kg	2100
Weeks 7–8	3	10	75% 1RM	3 sets * 10 reps * 75 kg	2250
**Endurance**					
Weeks 1–2	3	20	30% 1RM	3 sets * 20 reps * 30 kg	1800
Weeks 3–4	3	20	35% 1RM	3 sets * 20 reps * 35 kg	2100
Weeks 5–6	3	20	40% 1RM	3 sets * 20 reps * 40 kg	2400
Weeks 7–8	3	20	45% 1RM	3 sets * 20 reps * 45 kg	2700

RM: repetition maximum.

To guarantee safety, tolerability will be assessed in each session using a numerical rating scale for pain (NRS, scale 0–10) or, where appropriate, physical examination. For NRS > 5, training load is modified. PTs may provide tailored instructions during sessions, following the same procedure for both groups.

The home-based exercises are initially taught at Reade and consist of bodyweight and resistance bands exercises. Exercise selection is the same across groups. However, the strength group uses bands with greater resistance. Additionally, repetition counts and rest intervals mirror those used in supervised sessions. Patients are instructed to target an intensity of 7–8 on a 10-point physical exertion scale. To ensure adherence to the intended intensity level, patients are asked to be aware of effort using easily measurable physiological indicators such as breathing rate, perspiration, and muscle soreness. Exercises are regularly evaluated and adjusted as needed by the treating physiotherapist.

#### Additional intervention.

As part of regular clinical care all patients will receive two one-hour educational sessions from an occupational therapist on improving their capacity, the balance between physical and mental load, and joint-protection. Where indicated by the supervising rehabilitation physician, participants may also receive psycho-education, support from a social worker, or podiatry.

### Outcome measures

Fig 1 lists all descriptive data, primary and secondary outcomes, and other measures (e.g., treatment adherence and process measures).

The primary outcome is isokinetic muscle strength (*peak torque)* assessed using an isokinetic dynamometer Biodex System (Enknee Enraf-Nonius, the Netherlands)). **Peak torque** will be measured in both upper legs using knee extension test. Participants will first perform a familiarization trial. Subsequently, they will complete three maximal-effort repetitions at an angular velocity of 60°/s to assess the isokinetic strength of the quadriceps for each leg. The average torque (in Newton-meters, Nm) produced by the quadriceps per leg will be calculated and normalized to body weight (Nm/kg). For analysis, values from the index knee (i.e., the most affected knee) will be used [36]. In addition to absolute peak torque (Nm), relative muscle force will be assessed by calculating torque relative to physiological cross-sectional area (PCSA) as derived from 3D ultrasound (Nm/cm^2^). This provides an indication of muscle quality (i.e., force-generating capacity per unit muscle area).

Measurements will be performed at the start and end of the exercise intervention (except the muscle biopsy, which will be only taken prior to the intervention).

Secondary outcome measures include muscle endurance, and i) muscle morphology, (ii) mitochondrial function, and (iii) systemic inflammation (see Table 4). These parameters will be assessed using the Six minute Walk test (6MWT), an isokinetic dynamometer, 3D ultrasound (3D US), high-resolution respirometry (HRR), and blood-based biomarker analysis (Multiplex/ELISA), respectively and will be described in detail.

**Table 4:** Overview of secondary outcome measures, examples of measurements, related pathways relevant to the C4M study and the techniques to be applied.

Secondary Outcome Measure	Examples of read-outs	Related Pathway relevant to C4M	Technique to be applied
(i) **Muscle morphology**	• muscle belly length (ℓm); muscle fascicle length (ℓfasc); physiological cross-sectional area (PCSA); tendon length (ℓt); and muscle-tendon complex length (ℓm + t)	Muscle plasticity	3DUS
(ii) **Muscle mitochondrial function**	• Basal respiration; Leak respiration; NADH-linked respiration, Succinate-linked respiration, Maximal Oxidative Capacity; Maximum uncoupled respiration	Energy production (/ oxidative capacity)	high-resolution respirometry
**iii) Blood biomarkers**	• IL-6; IFN-γ; IL-17; IL2; IL-4; IL-10 and TNF-α	Systemic inflammation	Multiplex/ ELISA

Muscle endurance: The 6MWT will be used to measure functional endurance. Participants are instructed to walk as far as possible along a 30-meter walkway for six minutes. The total distance covered (meters) will be recorded as an indicator of functional endurance [37]. Muscle specific endurance will be assessed by using an isokinetic dynamometer Biodex System**®**, using the same setup as for peak torque. Resistance will be set at 40–60% of the participant’s average peak torque to ensure submaximal loading. Participants will perform repeated concentric knee extensions until torque output drops by 15% from the initial 40–60% level. The total number of repetitions completed before this decline is recorded.

i) Muscle morphology: 3D US (Affiniti 30 Ultrasound system of Philips, Bothell US) of the vastus lateralis muscle of the index knee will be used to quantify volume, muscle belly length (ℓm), muscle fascicle length (ℓfasc), physiological cross-sectional area (PCSA), tendon length (ℓt), and muscle-tendon complex length (ℓm + t) [38] of the vastus lateralis. Muscle volume is measured between the origin and distal end of the muscle belly using manual segmentation of the anatomical cross-sections and interpolation in custom software. Average fascicle length (ℓfasc) and pennation angles (αfasc) are estimated. A pennation angle is the angle between the longitudinal axis of the entire muscle and its fibers. PCSA is calculated by dividing muscle volume by ℓfasc.
ii) Mitochondrial function: HRR will be used to measure mitochondrial energy production (i.e., mitochondrial oxidative capacity) with the Oxygraph-2k high-resolution respirometer (Oroboros Instruments, Innsbruck, Austria), [39]. In brief, mitochondrial function will be assessed with permeabilized muscle fibers originating from the muscle biopsy. The biopsy will be performed at the mid-section of the vastus lateralis of the quadricep muscle under local anesthesia (2–3 mL of 1% xylocaine), using a Bergström needle modified for manual suction. A small incision (~1 cm) will be made through which the biopsy is retrieved, and closed with skin glue or, if needed, sutures (Donati stitch). Part of the biopsy will be mechanically separated under a microscope to expose individual muscle fibers, followed by gentle teasing with fine forceps to improve substrate accessibility. This physical permeabilization step ensures that the sarcolemma is disrupted while preserving mitochondrial structure and function, allowing direct measurement of mitochondrial respiration using the Oxygraph system. Fibers will be transferred to the Oxygraph chambers and mitochondrial oxygen consumption will be continuously measured while performing a substrate-inhibitor-uncoupler-titration protocol. This protocol involves sequential addition of glutamate, malate, and pyruvate to assess complex I supported leak respiration, followed by ADP to stimulate coupled oxidative phosphorylation (OXPHOS) via complex I (NADH-linked), cytochrome *c* to test for outer mitochondrial membrane integrity, succinate to assess complex II supported respiration (Succinate-linked), FCCP to reveal total uncoupled electron transport system (ETS) capacity, rotenone to inhibit complex I, and finally antimycin A to inhibit complex III and reveal residual oxygen consumption. Systemic inflammation: blood will be withdrawn for investigation of blood-biomarkers for systemic inflammation (IL-6; IFN-γ; IL-17; IL-2; IL-4; IL-10 and TNF-α). Serum will be sampled from all participants and analyzed with multiplex cytokine analysis or ELISA (BD™ Cytometric Bead Array (CBA) Human Th1/Th2/Th17 CBA Kit, BD Biosciences, San Jose, CA, USA) to identify the cytokines that are differentially expressed in the RA, OA, and SARC patients. The following general inflammation markers will be assessed in addition to that: C-reactive protein (CRP) and erythrocyte sedimentation rate (ESR).

Other measures: To enable in-depth characterization of the study participants the following variables will be collected.

a. Patient characteristics: Sociodemographic and lifestyle characteristics will be assessed using a standardized questionnaire, including age, gender, height, weight, educational level, employment status, marital status, smoking and alcohol use (including duration), symptoms, and use of walking devices. Comorbidity will be assessed using the Cumulative Illness Rating Scale (CIRS) [40], administered through a semi-structured interview. Information on current medication use will be obtained from the participant’s medical record. Protein intake will be assessed using a 24-h dietary recall conducted by a trained researcher. Protein intake (g/day) will be calculated using the Netherlands Nutrition Centre database.
b. Anthropometrics: Body composition will be assessed using direct-segmental multi-frequency bio-electrical impedance analysis (DSM-BIA) (Biospace Co, Korea). DSM-BIA has been validated for assessing segmental and whole-body composition against dual energy X-ray absorptiometry (DXA) [41]. Muscle mass is expressed as SMM (kg), SMM index (SMI, kg/m^2^) by dividing SMM (kg) by height squared (m^2^) [30], relative SMM (%) by dividing SMM (kg) by body weight (kg)*100, ALM/height^2^ (kg/m^2^) by dividing ALM (kg) by height squared (m^2^) and fat free mass (%).
c. Disease characteristics: diagnosis will be obtained from the participant’s medical record; Radiologic severity of osteoarthritis will be assessed using Kellgren and Lawrence grade (K&L) [42].
d. Performance: handgrip strength will be assessed with a JAMAR**®** Dynometer (Patterson Medical, UK) [43,44]. Participants will be seated with elbows flexed at 90 degrees, shoulders adducted and forearms in a neutral position without support. They will be instructed to squeeze the dynamometer maximally three times per hand, alternating between the right and left sides. The highest recorded value (kg) will be reported; Short Physical Performance Battery (SPPB test) [45]. The SPPB assesses lower extremity function through three components i) Balance Test: Participants perform progressively challenging standing positions (feet together, semi-tandem, and full tandem stance) for up to 10 seconds each. ii) Gait Speed Test: Walking speed is measured over a 4-meter course. iii) Chair Stand Test: Participants complete five timed chair rises without using their arms for support. The time to complete the five repetitions will be recorded in seconds. The total SPPB score (0–12) reflects overall lower limb function, with higher scores indicating better performance.
e. Patient-Reported Outcome Measures (PROMs) will be measured with online questionnaires: Numeric Rating scale pain (0–10) [46], PROMIS® short forms regarding fatigue (8a) version 1.0, physical function (8b) version 2.0, ability to participate in social roles and activities (8a) version 2.0 [47]. FitMáx© survey [48] is a questionnaire consisting of three single answer questions, about the maximum capacity of walking (scale from 0–13), climbing stairs (scale 0–10) and cycling (scale 0–11). By adding the participant’s sex, age and BMI, the FitMáx© model estimates the maximum oxygen uptake, expressed in ml/kg/min [48,49].

EuroQol- 5 Dimension (EQ5D) questionnaire is used to assess five domains (mobility, self-care, usual activities, pain/discomfort, anxiety/depression) and includes a visual analogue scale (VAS) for self-rated health status [50].

### Treatment adherence

Treatment adherence (number of sessions attained) will be prospectively documented by the treating PT in a logbook. For the home-based exercise program, participants will record number of training sessions and perceived training intensity on a Borg-scale [51] in a logbook.

### Other process measures

Feasibility: The drop-out rate, defined as the proportion of participants who discontinue their participation before completing the study protocol, will be determined by the number of participants who provide informed consent but fail to complete the follow-up assessments at the predefined end point (e.g., 8 weeks). Reasons for drop-out will be recorded where available (e.g., adverse events, loss of motivation).User satisfaction with Reforter™ system will be assessed with the Net Promoter Score (NPS) [52], a single-item questionnaire asking participants to rate the likelihood of recommending the Reforter™ system to others on a scale from 0 (not at all likely) to 10 (extremely likely).

### Adverse events

Adverse events or side effects of the training program will be prospectively documented by the treating PT in a logbook.

### Intervention fidelity

To monitor treatment fidelity, physiotherapists will complete customized semi-structured notes in a logbook for each treatment session. The research staff will regularly review these notes to ensure that the intervention is delivered according to the study protocol, including appropriate progression in exercise intensity.

### Trial sample size

This study was designed as an exploratory mechanistic trial because no previous studies have compared strength and endurance exercise in patients with RA, OA or SARC. Consequently, no reliable disease-specific between-group effect size was available to perform a formal a priori sample size calculation for the primary comparison. The target sample size was therefore determined pragmatically, balancing the feasibility of the extensive mechanistic assessments (including muscle biopsies, mitochondrial respirometry and inflammatory biomarker analyses) with the aim of including as many participants as possible within the available resources and study period. A total of 69 patients will be included, taking into account a 15% dropout rate, 60 patients will be analysed of which 30 will be randomized to the strength group and 30 to the endurance group, stratified by diagnosis (OA, SARC and RA) and gender. This resulted in a target sample size of 69 participants (23 per disease group). The study is intended to estimate intervention effects, variability and mechanistic associations to inform the design of future adequately powered confirmatory studies. The study is not powered for separate confirmatory analyses within each disease group.

### Data management

Each participant will be assigned a unique study identification code to ensure confidentiality. Personal identifying information will be stored separately from research data and will only be accessible to the principal investigator and authorized members of the research team. All study data will be collected and processed using this coded identifier.

Clinical data will be collected by a trained researcher at Reade and recorded in electronic case report forms in Castor EDC at Amsterdam UMC. Data related to muscle biopsies and laboratory analyses will be collected and stored at Amsterdam UMC. Treatment adherence, training intensity, and adverse events will be recorded by the treating physiotherapists in standardized logbooks. These data will subsequently be entered into the electronic case report forms in Castor EDC by a member of the research team. All study data will be stored on secure, password-protected institutional servers at Amsterdam UMC and Reade, in accordance with institutional data protection policies.

Data quality will be ensured through predefined data entry checks and periodic monitoring procedures. Source data verification will be performed on a sample of records and focused on key study variables, informed consent documentation, and protocol adherence.

Study data will be retained for a minimum of 15 years after completion of data collection, in line with institutional and regulatory requirements. De-identified data underlying the findings of this study will be made available upon reasonable request, subject to ethical approval and data protection regulations.

### Data analysis plan

Descriptive statistics will be used to analyze the participant characteristics including sociodemographic variables (e.g., age, sex, educational level, living situation, smoking, alcohol consumption), clinical factors (e.g., diagnosis, comorbidities, medication use), anthropometric measures (e.g., BMI, muscle mass), and baseline outcome variables. Muscle-related characteristics will be grouped and described according to their physiological domain (e.g., muscle morphology, muscle strength, mitochondrial function and blood biomarkers). Analyses will be performed for baseline and baseline compared to follow-up (delta muscle strength). Analyses will encompass calculations of means (SD)/ median [IRQ], scatterplots and covariance matrices, stratified by diagnosis and gender and with respect to main study endpoint.

#### 1. Primary outcome (isokinetic muscle strength).

The effect of exercise type (strength vs. muscle endurance exercise) on isokinetic muscle strength (pre- vs. post-intervention) will be assessed using linear mixed models. Models will include fixed effects for exercise group, time (pre/post), exercise group, disease type, and gender. The time × exercise group interaction will be included to estimate differential changes in muscle strength between intervention groups. Random effects will account for individual variability.

#### 2. Secondary outcome (Associations between muscle characteristics and exercise response).

i) Linear regression analyses will be conducted for each characteristic, e.g., muscle morphology (marker: DSM-BIA, physiological cross-sectional area and pennation angle), mitochondrial function (marker: Basal respiration; Leak respiration; NADH-linked respiration, Succinate-linked respiration, Maximal Oxidative Capacity; Maximum uncoupled respiration), and inflammation (markers: IL-6; IFN-γ; IL-17; IL2; IL-4; IL-10 and TNF-α). The change in muscle strength (T1 – T0) will be the dependent variable, and each muscle characteristic will serve as the independent variable.
ii) To explore whether associations between muscle characteristics and exercise response differ by exercise modality, models will be extended by including exercise group and interaction terms (muscle characteristic × exercise group). Given the exploratory nature of the study and the limited sample size, these analyses are intended to identify potential signals rather than provide definitive evidence of effect modification. Interaction estimates will be interpreted cautiously, with emphasis on estimated effect sizes and 95% confidence intervals rather than statistical significance testing.
iii) Adjusting for covariates. All models will be adjusted for the minimization factors (gender and disease type) and prespecified covariates (age and BMI). Given the exploratory nature of the study and the available sample size, the number of covariates will be restricted to reduce the risk of model overfitting..
iv) Aforementioned steps will be repeated with other clinical outcomes (isokinetic muscle endurance, 6MWT, SPPB physical function and pain) as the dependent variables to address the secondary objective of the study.

Missing data will be addressed using the Multivariate Imputation by Chained Equations (MICE) method when data were assumed to be Missing Completely at Random (MCAR) or Missing at Random (MAR). Variables with substantial missingness (>30%) or data deemed Missing Not at Random (MNAR) will be excluded. Analyses on imputed data will be compared with the original dataset to assess consistency.

Analyses will be based on the intention-to-treat principle (ITT), in which data of all participants will be analyzed according to group assignment. In addition, per-protocol analysis will be performed including only those participants that performed 80% or more sessions.

### Patient and public involvement

A patient research partner provided input into the research question, the study protocol and the patient information letter. Three physiotherapists provided specific input on the training protocol.

## Discussion

This protocol paper describes the rationale of a two-arm parallel-group exploratory trial comparing muscle endurance versus strength exercises in patients with RA, OA, and sarcopenia. The study was motivated by the clinical need to better understand how distinct mechanisms of muscle weakness, arising from autoimmune, degenerative, or age-related conditions, shape responses to exercise training. Although exercise is widely acknowledged as effective for improving muscle strength and function [7,53,54], the optimal training strategy for specific patient groups remains unclear [3,17–19]. Findings may provide insights into the molecular and cellular mechanisms underlying muscle weakness, including pathways related to muscle morphology, mitochondrial function, and systemic inflammation. Such knowledge may contribute to the development of tailored exercise interventions to optimize therapeutic outcomes.

The parallel-group design with stratified randomization was chosen to systematically compare strength- and endurance-focused exercises and to explore mechanistic associations with muscle characteristics. Several methodological considerations are relevant. First for the RA group, a broad range of disease activity levels was included to explore whether disease activity influences exercise outcomes. This choice may increase heterogeneity within the RA sample but was considered acceptable, as it improves the feasibility of recruitment and allows examination of how inflammatory disease activity may modulate exercise responses. Second, primary and secondary outcomes were selected to balance clinical relevance and mechanistic depth. Isokinetic muscle strength was prioritized given its importance for daily functioning [55], while secondary measures (3D ultrasound morphology, mitochondrial respirometry, inflammatory biomarkers) provide mechanistic context. Additional assessments of muscle endurance, functional performance, patient-reported outcomes, and quality of life extend the clinical scope of the trial. However, the assessment of muscle endurance presents challenges. Although VO_2_-max testing is the gold standard, logistical constraints made it unfeasible in this study. We therefore use the 6MW to measure functional endurance and an isokinetic Biodex®-based fatigue test to measure muscle specific endurance. The 6MWT is validated and widely applied [37], but no standardized test exists for isokinetic muscle endurance; the Biodex®-based test should therefore be considered exploratory.

A limitation of this trial is that the sample size, while adequate for the exploratory objectives of the study, limits the estimation of precision of subgroup-specific and interaction effects. The heterogeneity in disease pathology and baseline muscle characteristics further complicates the interpretation of disease-specific analyses. Consequently, multivariable and interaction analyses should be regarded as hypothesis-generating rather than confirmatory, with emphasis placed on effect size estimates and confidence intervals rather than formal hypothesis testing. Nevertheless, the inclusion of 69 participants across three diagnostic groups will allow estimation of variability in muscle characteristics and provide preliminary effect size estimates to inform the design of future confirmatory studies.

Despite these limitations, the exploratory design is justified by absence of studies directly comparing strength and endurance training across RA, OA, and SARC. Importantly, the mechanistic assessments will provide new insights into pathways of muscle morphology, mitochondrial function, and inflammation. The findings may help clarify whether these characteristics are associated with variability in exercise response and may guide future mechanistic studies using more advanced laboratory techniques, such as transcriptomic, proteomic, or immunohistochemical analyses.

In summary, this exploratory trial will investigate whether muscle morphology, mitochondrial function, and inflammatory status are associated with variability in response to strength and endurance exercise in patients with RA, OA, and sarcopenia. The findings may provide preliminary evidence to guide future mechanism-based exercise trials and contribute to the development of more targeted rehabilitation strategies for patients with muscle weakness.

## Supporting information

S1 File Supervised exercises. (DOCX)

S2 Checklist SPIRIT 2025 checklist. (DOCX)

## Abbreviations

BMI: Body Mass Index
CIRS: Cumulative Illness Rating Scale
DXA: Dual-energy X-ray absorptiometry
DSM-BIA: direct-segmental multi-frequency bio-electrical impedance analysis
EQ5D: EuroQol- 5 Dimension
IC: informed Consent
ITT: intention-to-treat
ℓfasc: muscle fascicle length
ℓm: muscle belly length
ℓm + t: muscle-tendon complex length
ℓt: tendon length
NRS: Numeric Rating Scale
MAR: Missing at Random
MCAR: Missing Completely at Random
MICE: Multivariate Imputation by Chained Equations
MNAR: Missing Not at Random
OA: Osteoarthritis
PCSA: physiological cross-sectional area
PROMs: Patient-Reported Outcome Measures
PT: physiotherapist
RA: Rheumatoid Arthritis
RM: repletion maximum
SARC: Sarcopenie
6MWT: Six Minute Walktest
SPPB: Short Physical Performance Battery
VAS: visual analogue scale
