# Supplementary material for: Effects of exercise on muscle strength and characteristics in rheumatic diseases and sarcopenia: Protocol for the Care for Muscle (C4M) Study
Source: PLoS One. 2026 Sep 9;21(9):e0356181. doi: 10.1371/journal.pone.0356181 (PMC13557349; doi:10.1371/journal.pone.0356181)
Supplement: Supplementary file 1 [file pone.0356181.s001.docx]

| S1 Supervised exercises | | | | | |
| --- | --- | --- | --- | --- | --- |
| Muscle group | | **Exercise** | **Starting position and**  **safety instructions** | **Exercise instructions** | |
|  |  |  |  | **Strength protocol** | **Endurance protocol** |
|  |  |  |  | *3x 10 repetitions* | *3x 20 repetitions* |
| Functional / quadriceps | | Leg press (Machine) | Sit in leg press machine. Feet hip-width on platform, knees flexed. Keep hips stable and back neutral during the exercise. | Extend legs fully (but keep knees soft). Slowly flex knees and control on the way back to the starting position. Weight is set to 60% of 1RM and built up to 75% of 1RM over the 8 weeks. | Extend legs quickly without losing tension. Flex knees and come back to the starting position. ROM may be reduced to maintain high pace. Weight is set to 30% of 1RM and built up to 45% of 1RM over the 8 weeks. |
|  | | Box squat | Stand upright with knees slightly bend and feet shoulder-width apart. Make sure spine is neutral, core is activated, knees do not fall inwards. | Slowly move downwards. When knees are ±60-80 degrees bent, push back up. Over time, ROM may be increased. Either a weighted vest or dumbbells are used to achieve 60-75% of 1RM. | In high tempo, move downwards and come back up when knees are ±40 degrees bent. Over time, ROM may be increased. No extra weight is added. |
|  | | Split squat | Sit upright, take one step forward so one foot is in front of the other, keep feet shoulder-width apart. Keep weight on heel of the front foot during the movement. Make sure the front knee does not fall inwards. | Slowly move downwards. Once front knee is ±60 degrees bent, slowly come back up. Over time, ROM may be increased. Either a weighted vest or dumbbells are used to achieve 60-75% of 1RM. | In high tempo, move downwards. Quickly push back up when front knee is ±40 degrees bent. Over time, ROM may be increased. No extra weight is added. |
|  | | Leg extension (Reforter™) | Sit looking away from Reforter™. Ankle strap around ankle, leg relaxed and flexed. Focus on the upper leg and stay upright. | Explosively extend knee. On the way back, resist the resistance of the Reforter™ and move foot down in a slow and controlled manner.  Reforter™ settings: two-way isokinetic strength, at very low speed. | Kick up foot until knee is extended. When going back to starting position, follow the pace of the Reforter™ while feeling the resistance.  Reforter™ settings: two-way isokinetic endurance, at very high speed. |
| Glutes | | Hip abduction (Machine) | Sit in abductor machine, legs are together. Avoid jerking and keep the spine neutral. | Abduct legs by contracting the glutes. Control the motion. When legs are apart, hold for a second before slowly bringing the knees back in. | Abduct legs by contracting the glutes at a high speed. Come straight back to starting position. |
| Back | | Lat pulldown (Machine) | Sit in the machine, facing the weight stack. Hands are wide on bar above head. Elbows are extended but still soft (not locked). | Bring bar to upper chest in controlled pull, keep elbows to the side. Hold for a second before moving back to the starting position in a slow and controlled manner. | Bring bar to upper chest. Keep elbows to the side. Come straight back to starting position while keeping core engaged. |
|  | | Seated row (Reforter™) | Sit in front of Reforter™, facing the machine. Hands in front, arms extended. Keep elbows tucked in during the movement. | Bring handle towards the umbilicus. Hold for a second before moving back to the starting position. Try and resist the resistance of the Reforter™ and bring the bar back to its starting position as slowly as possible.  Reforter™ settings: two-way isokinetic strength, at very low speed. | Bring handle to the umbilicus in the pace indicated by the Reforter™. Move straight back to the starting position. When going back to starting position, follow the pace of the Reforter™ while feeling the resistance.  Reforter™ settings: two-way isokinetic endurance, at very high speed. |
| Arms | | Biceps curl (Reforter™) | Stand in front of Reforter™, facing the machine. Arms are relaxed with hands shoulder-width apart facing upwards, holding the bar in front of hips. Keep elbows tucked in during the movement. | Contract upper arm (biceps) to bring bar towards the chest. Hold for a second and bring handle back to its starting position as slowly as possible, resisting the pull of the Reforter™.  Reforter™ settings: two-way isokinetic strength, at very low speed. | Contract upper arm (biceps) to bring bar towards the chest. Move straight back to the starting position and follow the pace of the Reforter™ while feeling the resistance.  Reforter™ settings: two-way isokinetic endurance, at very high speed. |
|  | | Triceps pushdown (Reforter™) | Stand in front of Reforter™, facing the machine. Hands are shoulder-width apart facing downwards, holding the bar in front of chest. Keep elbows tucked in during the movement. | Push the bar down towards the hips by contracting the triceps. Bring handle back to its starting position as slowly as possible, resisting the pull of the Reforter™.  Reforter™ settings: two-way isokinetic strength, at very low speed. | Push the bar down towards the hips by contracting the triceps and go straight back to starting position. Follow the speed indicated by the Reforter™ while feeling the resistance.  Reforter™ settings: two-way isokinetic endurance, at very high speed. |
| Homebased exercise | | | | | |
| Lower body | Box Squat | | Starting position is as described above. No extra weight is added. | Move downwards in a slow and controlled manner. When a (front) knee angle of ±60 degrees is achieved, hold for a second and move back up. Build up to ±80 degrees over the course of the program. | At a high but maintainable tempo, move downwards. When a (front) knee angle of ±50 degrees is achieved, directly move back up. Build up to ±70 degrees over the course of the program. |
|  | Split squat | | Starting position is as described above for lunge, but with one hand on a support surface such as a table. | Slowly move downwards. Once front knee is ±60 degrees bent, slowly straighten knees again. Over time, ROM may be increased. Dumbbells or other home weight can be  used to achieve 60-75% of 1RM. | In high tempo, bend both knees simultaneously, then quickly push back up when front knee is ±40 degrees bent. Over time, ROM may be increased. No extra weight is added. |
|  | Crab walk  (Theraband) | | The Theraband is tied into a circle and wrapped around the lower legs, slightly below the knees. Standing up, feet are shoulder width apart and knees are in line with feet. | From the starting position, bend knees to approximately 60°. Keep constant tension on the Theraband. Take slow, controlled side steps of about one foot-length while maintaining the semi-squat position and alignment of knees and feet. Every two weeks, a slightly stiffer Theraband is used to increase resistance. Use them in the following order: yellow-red-green-blue | The yellow Theraband is used for this exercise. From the starting position, bend knees slightly (±30°) and maintain a semi-squat position. Perform continuous side steps at a moderate tempo. Ensure knees remain aligned with the feet and avoid inward collapse. Use the yellow Theraband for this exercise. Over the weeks, ROM and pace may be increased after consultation with the PT. |
| Upper body | Seated row (Theraband) | | Starting position is as described above, except we stand in front of a doorknob or other handle. A Theraband is wrapped around the handle at the middle. Each hand holds one end of the Theraband. During the exercise, keep core engaged and elbows tucked in. | Pull hands towards the belly, at umbilical height. When shoulder blades are retracted, hold for a second before returning to starting position. Every two weeks, a slightly stiffer Theraband is used to increase resistance. Use them in the following order: yellow-red-green-blue | The yellow Theraband is used for this exercise. Pull hands towards the belly, at umbilical height. When shoulder blades are retracted, immediately return to starting position. Over the weeks, ROM and pace may be increased after consultation with the PT. |
|  | Bicep curl (Theraband) | | Starting position is as described above, except we now stand on the middle of the theraband. Each hand holds one end of the theraband. During the exercise, keep core engaged and elbows tucked in. | Contract upper arm (biceps) to bring fists towards the chest. Hold for a second and return to starting position. Every two weeks, a slightly stiffer theraband is used to increase resistance. Use them in the following order yellow-red-green-blue | The yellow theraband is used for this exercise. Contract upper arm (biceps) to bring fists towards the chest. Move straight back to the starting position at a maintainable but high pace. Over the weeks, ROM and pace may be increased after consultation with the PT. |
|  | Tricep curl (Theraband) | | Starting position is as described above, except a Theraband is hung over a door. Each hand holds one end of the Theraband. During the exercise, keep core engaged and elbows tucked in. | Push fists down towards hips by contracting the triceps. Hold for a second before coming back to starting position in a slow and controlled manner. Theraband is used to increase resistance. Use them in the following order: yellow- red-green-blue | The yellow Theraband is used for this exercise. Push fists down towards hips by contracting the triceps at a maintainable but high pace. Move straight back to the starting position. Over the weeks, ROM and pace may be increased |
