# Supplementary material for: Effects of exercise on muscle strength and characteristics in rheumatic diseases and sarcopenia: Protocol for the Care for Muscle (C4M) Study
Source: PLoS One. 2026 Sep 9;21(9):e0356181. doi: 10.1371/journal.pone.0356181 (PMC13557349; doi:10.1371/journal.pone.0356181)
Supplement: Supplementary file 3 [file pone.0356181.s004.docx]

**Response to Reviewers’ Comments**

**Manuscript title:** *Effects of exercise on muscle strength and characteristics in rheumatic diseases and sarcopenia: Protocol for the Care for Muscle (C4M) Study*

Dear Dr. Akaras,

We sincerely thank the editor and the reviewers for their careful assessment of our manuscript and for their valuable comments and suggestions. Their feedback has helped us to improve both the clarity and the scientific quality of the manuscript. We have addressed each comment in detail below and revised the manuscript accordingly. All changes are indicated in the responses and in the revised manuscript.

**Response to Academic Editor’s Comments**

Comment 1. Manuscript formatting and PLOS ONE style requirements

*Response: We have revised the manuscript according to the PLOS ONE formatting requirements and ensured that the file naming conventions and formatting guidelines have been followed.*

Comment 2. Funding statement

Response and author action: *We have removed all funding-related text from the manuscript, including the Acknowledgments section, as requested. The Funding Statement provided in the online submission system is correct and does not require any amendments. The updated Funding Statement is also included in the cover letter.*

Comment 3. Ethics statement

*Response and author action: We have removed the ethics statement from all sections except the Methods section, where it is now appropriately reported.*

Comment 4. Figure 1 caption

*Response and author action: We have added a caption for figure 1 as requested*

5. If the reviewer comments include a recommendation to cite specific previously published works, please review and evaluate these publications to determine whether they are relevant and should be cited. There is no requirement to cite these works unless the editor has indicated otherwise. 

**Response to Reviewers’ Comments**

We sincerely thank the reviewers for their careful evaluation of our manuscript and for their constructive comments and suggestions. We have addressed each comment below and revised the manuscript accordingly.

Reviewer #1: The authors intend to explore the association between muscle characteristics and exercise outcomes in RA, OA, and sarcopenia through an exploratory trial with 69 patients allocated to two arms (endurance and muscle strength exercises).

1. Line 35. The authors claimed 69 participants to be sufficient to capture variability in muscle characteristics. Is there any evidence or reasoning to support this argument? Considering 3 disease groups and 2 intervention groups, each analytical group will be only around 11 participants!

*Response: Thank you for this important comment. We agree that the original wording suggested a level of statistical justification that was not adequately supported. The study is exploratory and was not powered for disease-specific subgroup analyses or interaction effects.*

*We agree that further clarification of the sample size rationale is warranted. The sample size of 69 participants was primarily chosen to ensure representation of the three clinical populations (RA, OA and sarcopenia) while maintaining feasibility for the extensive mechanistic assessments, including muscle biopsies and high-resolution respirometry. The study was designed as an exploratory mechanistic trial and the target sample size was determined pragmatically, balancing the feasibility of the extensive mechanistic assessments with the need to obtain sufficiently precise estimates of variability and intervention effects for future confirmatory studies.*

*As no studies have compared strength and endurance exercise in patients with RA, OA or sarcopenia, no reliable disease-specific between-group effect size was available to perform a formal a priori sample size calculation for the final study design. A convenient and feasible sample of a total of 69 patients will be included, taking into account a 15% dropout rate, 60 patients will be analysed of which 30 will be randomized to the strength group and 30 to the endurance group, stratified by diagnosis (OA, SARC and RA) and gender.*

*To provide additional context, we performed a post hoc evaluation using effect sizes reported in meta-analyses in healthy adults (Lopez P et al, Med. Sci. Sports Exerc 2021), which provided supportive context regarding the feasibility of the selected sample size for the primary comparison between exercise modalities. We have clarified this rationale in the Methods.*

*Author action: We have revised the sample size section accordingly and now explicitly state that the study is intended to generate exploratory estimates and inform future adequately powered trials.*

*Adapted in the manuscript (Page 13, line 393-405):* *This study was designed as an exploratory mechanistic trial because no previous studies have compared strength and endurance exercise in patients with rheumatoid arthritis, osteoarthritis or sarcopenia. Consequently, no reliable disease-specific between-group effect size was available to perform a formal a priori sample size calculation for the primary comparison. The target sample size was therefore determined pragmatically, balancing the feasibility of the extensive mechanistic assessments (including muscle biopsies, mitochondrial respirometry and inflammatory biomarker analyses) with the aim of including as many participants as possible within the available resources and study period. A total of 69 patients will be included, taking into account a 15% dropout rate, 60 patients will be analysed of which 30 will be randomized to the strength group and 30 to the endurance group, stratified by diagnosis (OA, SARC and RA) and gender. This resulted in a target sample size of 69 participants (23 per disease group). The study is intended to estimate intervention effects, variability and mechanistic associations to inform the design of future adequately powered confirmatory studies. The study is not powered for separate confirmatory analyses within each disease group*

2. Line 228. Are peak power and torque the same or interchangeable? In the method for the primary outcome, it’s unclear which one will be analyzed as the primary outcome.

*Response: Thank you for identifying this inconsistency. Peak power and torque are distinct biomechanical parameters and should not be used interchangeably. Our intended primary outcome is isokinetic knee extension peak torque measured using the Biodex dynamometer and normalized for body weight (Nm/kg).*

*Author action: We have revised the abstract, methods, outcome description and statistical analysis sections to consistently refer to peak torque as the primary outcome throughout the manuscript.*

*In addition to changes in the method section, we specified the text of the measurement of mitochondrial function (Page 11, Line 307-312):*

*This protocol involves sequential addition of glutamate, malate, and pyruvate to assess complex I supported leak respiration, followed by ADP to stimulate coupled oxidative phosphorylation (OXPHOS) via complex I (NADH-linked), cytochrome c to test for outer mitochondrial membrane integrity, succinate to assess complex II supported respiration (Succinate-linked), FCCP to reveal total uncoupled electron transport system (ETS) capacity, rotenone to inhibit complex I, and finally antimycin A to inhibit complex III and reveal residual oxygen consumption.*

3. Line 403. Interaction is also among their interest. Will 69 participants be enough? What minimal detectable effect size can be identified with the proposed sample size? Considering the small sample size, will the effect be estimable?

*Response: We agree that the proposed sample size is not sufficient to robustly detect small-to-moderate interaction effects. The interaction analyses are exploratory and intended to investigate potential signals that may inform future confirmatory studies.* *This is consistent with the study design, as the selected sample size was intended to address the primary intervention comparison and exploratory mechanistic objectives rather than to detect subgroup-specific or interaction effects.*

*Author action:* Accordingly, we have *revised the statistical analysis section to clarify that interaction estimates will be interpreted cautiously, with emphasis on effect sizes and confidence int*ervals *rather than formal hypothesis testing.*

We specified the text for the analyses for primary and secondary outcome:

*Adapted in the manuscript (page 15, line 448-469)*

1. Primary outcome (isokinetic muscle strength):
The effect of exercise type (strength vs. muscle endurance exercise) on isokinetic muscle strength (pre- vs. post-intervention) will be assessed using linear mixed models. Models will include fixed effects for exercise group, time (pre/post), exercise group, disease type, and gender. The time × exercise group interaction will be included to estimate differential changes in muscle strength between intervention groups. Random effects will account for individual variability.

2. Secondary outcome (Associations between muscle characteristics and exercise response)
ii) To explore whether associations between muscle characteristics and exercise response differ by exercise modality, models will be extended by including exercise group and interaction terms (muscle characteristic × exercise group). Given the exploratory nature of the study and the limited sample size, these analyses are intended to identify potential signals rather than provide definitive evidence of effect modification. Interaction estimates will be interpreted cautiously, with emphasis on estimated effect sizes and 95% confidence intervals rather than statistical significance testing.

iii) Adjusting for covariates. All models will be adjusted for the minimization factors (gender and disease type) and prespecified covariates (age and BMI). Given the exploratory nature of the study and the available sample size, the number of covariates will be restricted to reduce the risk of model overfitting.

4. Minimization ensures balance on disease type and gender, but not other factors, such as BMI and so on. Will these factors be adjusted in the primary models? Please specify. If so, will the same size be enough to make the model estimable?

*Response: Thank you for this suggestion. In addition to the minimization factors (disease type and gender), age and BMI will be included as prespecified covariates in the regression models because of their known associations with muscle characteristics and training response.*

*Given the exploratory nature of the study and limited sample size, the number of covariates will be restricted to avoid model overfitting.*

*Author action: We have clarified this approach in the statistical analysis*

Adapted in the manuscript (page 15, line *467-469)*

5. Will analysis be conducted within each disease group? If not, will there be heterogeneity across disease groups?

*Response: The primary analyses will be conducted on the combined study population, with disease type included as a fixed effect in the models. The study is not powered for separate confirmatory analyses within each disease group.*

*Potential heterogeneity across disease groups will be explored through disease-specific descriptive analyses and exploratory interaction terms. Any disease-specific findings will be interpreted as hypothesis-generating.*

Reviewer #2: ABSTRACT:
*There is no an exact discussion, pls improve.

*Response: Thank you for this comment. We are not entirely sure how to interpret this remark, as the abstract already includes a Discussion section. We would appreciate further clarification regarding the specific aspects that the reviewer feels should be improved.*

Background:
the last sentence of the first paragraph:....correct inter-idividual to inter-individual

*Response and authors action:* Thank you. The spelling error has been corrected (page 4, line 77).

DISCUSSION:
Relatively moderate sample size is not a limitation. Remove this sentence. Actually your achieved power is %83 and %90 for two statistical analysis, and both power rates are in clinically acceptable limits. Below you will find your protocols of power analysis calculated by G-Power. You’d better write in a sentence the power and the calculated sample size in the methods section, and remove the limitation mentioned in the discussion section. Note: You can state small sample size as a limitation if the power is <%80.
Protocol of Power Analysis:
F tests - ANOVA: Fixed effects, omnibus, one-way (for group comparisons of the results of three groups: RA, osteoarthritis and Sarcopenia)
Analysis: A priori: Compute required sample size
Input: Effect size f = 0.4 (large, Cohen’s)
α err prob = 0.05
Power (1-β err prob) = 0.83
Number of groups = 3
Total sample size = 69
Actual power = 0.8365988

Protocol of Power Analysis:
4] -- Tuesday, May 12, 2026 -- 23:11:40
t tests - Means: Difference between two independent means (two groups) (for comparing the results of endurance exercise and muscle strength exercise groups)
Analysis: Post hoc: Compute achieved power
Input: Tail(s) = Two
Effect size d = 0.8 (large, Cohen’s)
α err prob = 0.05
Sample size group 1 = 35
Sample size group 2 = 34
Power (1-β err prob) = 0.9056359

*Response: We thank the reviewer for this helpful comment and for performing the G*Power calculations. These calculations were very helpful in performing our thoughts for determining the sample size. We agree that further justification of the selected sample size is warranted.*

*The proposed calculations are based on an assumed large intervention effect (Cohen's d = 0.8), for which no disease-specific evidence is currently available. No studies have compared strength and endurance exercise in patients with RA, OA or sarcopenia, and therefore no disease-specific between-group effect size was available to perform a formal a priori sample size calculation for the final study design.*

*This study was designed as an exploratory mechanistic trial rather than a confirmatory efficacy trial. Consequently, the target sample size of 69 participants was determined pragmatically, balancing the feasibility of the extensive mechanistic assessments (including muscle biopsies and mitochondrial analyses) with the aim of including as many participants as possible to obtain preliminary estimates of variability and effect sizes for future confirmatory studies. Hereby, taking into account a 15% dropout rate, 60 patients will be analysed of which 30 will be randomized to the strength group and 30 to the endurance group, stratified by diagnosis (OA, SARC and RA) and gender.*

*To place the selected sample size into context, we performed a post hoc evaluation using effect sizes reported in meta-analyses of resistance training in healthy adults, which consistently report small-to-moderate between-group effects (approximately Cohen's d = 0.3–0.4) for muscle strength outcomes (Lopez P et al, Med. Sci. Sports Exerc 2021). These findings provide reassurance that the planned sample size is likely to be adequate for the primary intervention comparison. However, this post hoc evaluation should be regarded as supportive rather than as an a priori justification of the sample size.*

*Author action: We agree that the wording of the limitation section could be improved. We have revised the discussion to clarify that the limitation relates not to detecting overall group differences, but rather to the ability to estimate subgroup-specific and interaction effects with precision. The study should therefore be regarded as exploratory and hypothesis-generating*

*Adapted in the manuscript (page 17, line 517-524)*

*A limitation of this trial is that the sample size, while adequate for the exploratory objectives of the study, limits the estimation of precision of subgroup-specific and interaction effects. The heterogeneity in disease pathology and baseline muscle characteristics further complicates the interpretation of disease-specific analyses. Consequently, multivariable and interaction analyses should be regarded as hypothesis-generating rather than confirmatory, with emphasis placed on effect size estimates and confidence intervals rather than formal hypothesis testing. Nevertheless, the inclusion of 69 participants across three diagnostic groups will allow estimation of variability in muscle characteristics and provide preliminary effect size estimates to inform the design of future confirmatory studies.*

Reviewer #3: The abstract requires one clear aim sentence, so does the introduction section.

*Response:* *We thank the reviewer for this comment. We agree that a clearly defined aim sentence is essential in both the abstract and the introduction. The aim of the study was already described in the submitted manuscript; however, we acknowledge that the formulation could be made more concise and explicit.*

*Author action:* *To further improve clarity, we have revised the wording of the aim sentence. The hypothesis statement has been retained as a separate sentence following the aim to clearly distinguish the study objective from the underlying rationale.*

*Adapted in the manuscript (Abstract page 2, line 29, Introduction page 4, line 99-101)*

*Abstract: This study aims to compare the effects of strength and endurance exercise and explore the association between muscle characteristics and exercise outcomes in patients with RA, OA and sarcopenia. We hypothesize that responses to endurance and strength exercises in patients with muscle weakness are influenced by intramuscular pathology including muscle morphology, mitochondrial function, and systemic inflammation, based on their disease pathology, potentially requiring personalized training schedules.*

*Introduction: Therefore, this study aims to compare the effects of strength and endurance exercise and explore the association between muscle characteristics and exercise outcomes in patients with RA, OA and SARC. We hypothesize that responses to endurance and strength exercises in patients with muscle weakness are influenced by intramuscular pathology including muscle morphology, mitochondrial function, and systemic inflammation.*

Please refrain from AI-generated phrases.

*Response:* *Thank you for this observation.*

Author action: *We carefully reviewed the manuscript and revised sections where the wording appeared overly generic or formulaic. We have ensured that the final manuscript reflects the authors’ own interpretation and scientific writing style.*

*In addition, we added a statement in the method section regarding the use of generative AI-assisted tools in the writing process* (Adaptations in the manuscript page 6, line 136-139)*:*

*“**During the preparation of this work, the authors used ChatGPT -5.5 to assist with grammar and language editing. After using this tool, the authors reviewed and revised the text as necessary and take full responsibility for the final content of the publication.*

Please pay attention to the use of tenses all throughout the text.

*Response and author action:* *Thank you for this observation. We carefully reviewed the manuscript and revised the use of tenses throughout the text where necessary.*

Each software and device should be given along with manufacturer and country of origin.

*Response and author action: We added the following manufacturer and country of origin of the following software and devices.*

*Adaptations in the manuscript page 9, 10, 11,12)*

- *Biodex system (Enknee Enraf-Nonius, the Netherlands)*
- *3D US (Affiniti 30 Ultrasound system of Philips, Bothell US)*
- *Multiplex assay / ELISA (BD™ Cytometric Bead Array (CBA) Human Th1/Th2/Th17 CBA Kit, BD Biosciences, San Jose, CA, USA)*
- *DSM-BIA**, Biospace Co, Korea*
- *Jamar (Patterson Medical, UK)*

Does each keyword exist in MeSH browser?

*Repsonse/author action: Thank you for this comment. We reviewed all keywords using the MeSH Browser. All selected keywords correspond to established MeSH terms. Following this review, we replaced "Muscle Weakness" with "Muscle Strength", as this term more accurately reflects the primary outcome and focus of the study.*

Exercise-related benefits include increased mitochondrial biogenesis/enzyme activity, reconditioning of immune/inflammatory pathways, decreased endoplasmic reticulum stress. Please cite relevant papers (PMID: 33793075) and provide a deeper insight to the topic for readers.

*Response: Thank you for this suggestion. We agree that exercise induces a broad range of biological adaptations beyond improvements in muscle strength and oxidative capacity. To better reflect the current understanding of exercise physiology, we expanded the background section by briefly describing additional exercise-induced adaptations, including mitochondrial biogenesis, modulation of inflammatory pathways, and reduced endoplasmic reticulum stress, while maintaining the focus of the introduction on the rationale for the present study.*

*Author action:* To provide additional context for readers, we have expanded the background section with a brief description of broader exercise-related adaptations, including mitochondrial adaptations, modulation of inflammatory pathways, and cellular stress responses, while maintaining the focus on the rationale and objectives of the present study

*Adapted in the manuscript Introduction page 4, line 72-75:*

*‘’Exercise training can counteract low muscle quantity and quality [11]. In addition to improvements in muscle strength and physical performance, exercise induces multiple biological adaptations, including improved mitochondrial function and enhanced oxidative capacity, modulation of inflammatory pathways, and improved cellular stress responses such as reduced endoplasmic reticulum stress.( PMID: 33793075)’’*

The age cut-off should be explained.

*Response: Thank you for this comment. We agree that the rationale for the upper age limit was insufficiently supported by the available literature.*

*Author action: We have removed the age cut-off from the eligibility criteria to avoid introducing an arbitrary restriction and have removed the corresponding discussion point from the Discussion section. This protocol amendment has been submitted to the Medical Ethics Review Committee (METC) for review. The manuscript has been revised accordingly (Adapted in the manuscript page 16, line 500-503).*

RA and OA are rheumatic diseases, Sarcopenia can be observed is a distinct entity which can also accompany these diseases. The methodology is questionable in this context.

*Response: Thank you for this important comment. We agree that RA, OA and sarcopenia are distinct clinical entities. However, they share the common clinical manifestations of muscle weakness and impaired physical functioning. Our rationale for including these populations is that they are expected to represent different biological pathways contributing to muscle weakness, including systemic inflammation, local joint-related processes, and age-related muscle degeneration. We have expanded the Introduction to clarify this rationale and to explain that the study aims to investigate whether variability in exercise response is associated with specific muscle characteristics rather than disease labels alone.*

*Author action: We added the following paragraph to the Introduction to clarify the rationale for including the three disease groups (Adaptations to the manuscript (lines 90-99, page 3, 4): Although RA, OA and SARC represent distinct clinical entities, they all present with reduced muscle strength and impaired physical functioning. The mechanisms underlying muscle weakness are, however, expected to differ between these conditions, ranging from chronic systemic inflammation in RA, to local joint-related and low-grade inflammatory processes in OA, and age-related muscle degeneration in sarcopenia. These differences in underlying muscle characteristics may contribute to the substantial inter-individual variability observed in response to exercise and highlight the need for a better understanding of mechanism-based exercise approaches*

"We aim to include 69 participants" How could you end up with this sample size?

*Author response: Thank you for this comment. As no data are available to estimate the expected difference between strength and endurance exercise in RA, OA or sarcopenia, a formal a priori sample size calculation for the primary comparison was not feasible. Therefore, the study was designed as an exploratory mechanistic trial and the target sample size was determined pragmatically, balancing the feasibility of the extensive mechanistic assessments with the need to obtain preliminary estimates of intervention effects and variability for future confirmatory studies. A sample of a total of 69 patients will be included, taking into account a 15% dropout rate, 60 patients will be analysed of which 30 will be randomized to the strength group and 30 to the endurance group, stratified by diagnosis (OA, SARC and RA) and gender. We have clarified this rationale in the Methods (Adapted in the manuscript page 13, line 392-405).*

Future insights could be added.

*Author action:* *We have changed future insights in the discussion* section (Adapted in the manuscript (page 17 line 534-536, 541-543):

*The findings may help clarify whether these characteristics are associated with variability in exercise response and may guide future mechanistic studies using more advanced laboratory techniques, such as transcriptomic, proteomic, or immunohistochemical analyses.*

The reference list could be updated and diversified.

*Response and author action: Thank you for this suggestion. We reviewed and updated the reference list by incorporating additional recent and relevant references, resulting in a more comprehensive and balanced overview of the current literature.*

DXA is the preferred abbreviation for Dual-energy X-ray absorptiometry

*Response and author action: We corrected the abbreviation for Dual-energy X-ray absorptiometry in the manuscript (*Adapted in the manuscript page 18, line 552).

Does each author meet the first two criteria of ICMJE?

*Response: Thank you for this comment. We confirm that all listed authors meet the ICMJE authorship criteria, including substantial contributions to the conception and design of the study and/or the development of the protocol, as well as drafting or critically revising the manuscript. All authors approved the final version of the manuscript and agree to be accountable for all aspects of the work.*
