# Supplementary material for: Effects of exercise on muscle strength and characteristics in rheumatic diseases and sarcopenia: Protocol for the Care for Muscle (C4M) Study
Source: PLoS One. 2026 Sep 9;21(9):e0356181. doi: 10.1371/journal.pone.0356181 (PMC13557349; doi:10.1371/journal.pone.0356181)
Supplement: Peer review history

# Peer review: Decision Letter 0

**DOI:** 10.1371/journal.pone.0356181.r001

**Authors:** Esedullah Akaras

1 Jun 2026

Dear Dr. de Rooij,

Thank you for submitting your manuscript to PLOS ONE. After careful consideration, we feel that it has merit but does not fully meet PLOS ONE’s publication criteria as it currently stands. Therefore, we invite you to submit a revised version of the manuscript that addresses the points raised during the review process.

Please submit your revised manuscript by Jul 16 2026 11:59PM. If you will need more time than this to complete your revisions, please reply to this message or contact the journal office at plosone@plos.org. When you're ready to submit your revision, log on to https://www.editorialmanager.com/pone/ and select the 'Submissions Needing Revision' folder to locate your manuscript file.

- A letter that responds to each point raised by the academic editor and reviewer(s). You should upload this letter as a separate file labeled 'Response to Reviewers'.
- A marked-up copy of your manuscript that highlights changes made to the original version. You should upload this as a separate file labeled 'Revised Manuscript with Track Changes'.
- An unmarked version of your revised paper without tracked changes. You should upload this as a separate file labeled 'Manuscript'.

If you would like to make changes to your financial disclosure, please include your updated statement in your cover letter. Guidelines for resubmitting your figure files are available below the reviewer comments at the end of this letter.

If applicable, we recommend that you deposit your laboratory protocols in protocols.io to enhance the reproducibility of your results. Protocols.io assigns your protocol its own identifier (DOI) so that it can be cited independently in the future. For instructions see: https://journals.plos.org/plosone/s/submission-guidelines#loc-laboratory-protocols. Additionally, PLOS ONE offers an option for publishing peer-reviewed Lab Protocol articles, which describe protocols hosted on protocols.io. Read more information on sharing protocols at https://plos.org/protocols?utm_medium=editorial-email&utm_source=authorletters&utm_campaign=protocols.

As the corresponding author, your ORCID iD is verified in the submission system and will appear in the published article. PLOS supports the use of ORCID, and we encourage all coauthors to register for an ORCID iD and use it as well. Please encourage your coauthors to verify their ORCID iD within the submission system before final acceptance, as unverified ORCID iDs will not appear in the published article. *Only*  the individual author can complete the verification step; PLOS staff *cannot*  verify ORCID iDs on behalf of authors.

We look forward to receiving your revised manuscript.

Kind regards,

Esedullah Akaras

Academic Editor

PLOS One

Journal Requirements:

When submitting your revision, we need you to address these additional requirements.

1. Please ensure that your manuscript meets PLOS ONE's style requirements, including those for file naming. The PLOS ONE style templates can be found at

https://journals.plos.org/plosone/s/file?id=wjVg/PLOSOne_formatting_sample_main_body.pdf and

https://journals.plos.org/plosone/s/file?id=ba62/PLOSOne_formatting_sample_title_authors_affiliations.pdf

2. Thank you for stating the following in the Acknowledgments Section of your manuscript:

“This trial is supported by funding from health holland Topconsortium voor Kennis en Innovatie (TKI). The funder has no role in conduct, analysis or reporting of this trial. Exercise devices will be available by Hapticlink company.”

We note that you have provided funding information that is not currently declared in your Funding Statement. However, funding information should not appear in the Acknowledgments section or other areas of your manuscript. We will only publish funding information present in the Funding Statement section of the online submission form.

Please remove any funding-related text from the manuscript and let us know how you would like to update your Funding Statement. Currently, your Funding Statement reads as follows:

“This trial is supported by funding from health holland Topconsortium voor Kennis en Innovatie (TKI). The funder has no role in conduct, analysis or reporting of this trial. Exercise devices will be available by Hapticlink company.”

Please include your amended statements within your cover letter; we will change the online submission form on your behalf.

3. Your ethics statement should only appear in the Methods section of your manuscript. If your ethics statement is written in any section besides the Methods, please delete it from any other section.

4. Please include a caption for figure 1.

5. If the reviewer comments include a recommendation to cite specific previously published works, please review and evaluate these publications to determine whether they are relevant and should be cited. There is no requirement to cite these works unless the editor has indicated otherwise.

[Note: HTML markup is below. Please do not edit.]

Reviewers' comments:

Reviewer's Responses to Questions

**Comments to the Author**

1. Does the manuscript provide a valid rationale for the proposed study, with clearly identified and justified research questions?

Reviewer #1: Yes

Reviewer #2: Yes

Reviewer #3: Partly

**********

2. Is the protocol technically sound and planned in a manner that will lead to a meaningful outcome and allow testing the stated hypotheses?

Reviewer #1: Yes

Reviewer #2: Yes

Reviewer #3: Partly

**********

3. Is the methodology feasible and described in sufficient detail to allow the work to be replicable?

Reviewer #1: Yes

Reviewer #2: Yes

Reviewer #3: Yes

**********

4. Have the authors described where all data underlying the findings will be made available when the study is complete?

The PLOS Data policy

Reviewer #1: Yes

Reviewer #2: Yes

Reviewer #3: No

**********

5. Is the manuscript presented in an intelligible fashion and written in standard English?

Reviewer #1: Yes

Reviewer #2: Yes

Reviewer #3: Yes

**********

Please use the space provided to explain your answers to the questions above and, if applicable, provide comments about issues authors must address before this protocol can be accepted for publication. You may also include additional comments for the author, including concerns about research or publication ethics.

You may also provide optional suggestions and comments to authors that they might find helpful in planning their study.

Reviewer #1: The authors intend to explore the association between muscle characteristics and exercise outcomes in RA, OA, and sarcopenia through an exploratory trial with 69 patients allocated to two arms (endurance and muscle strength exercises).

1. Line 35. The authors claimed 69 participants to be sufficient to capture variability in muscle characteristics. Is there any evidence or reasoning to support this argument? Considering 3 disease groups and 2 intervention groups, each analytical group will be only around 11 participants!

2. Line 228. Are peak power and torque the same or interchangeable? In the method for the primary outcome, it’s unclear which one will be analyzed as the primary outcome.

3. Line 403. Interaction is also among their interest. Will 69 participants be enough? What minimal detectable effect size can be identified with the proposed sample size? Considering the small sample size, will the effect be estimable?

4. Minimization ensures balance on disease type and gender, but not other factors, such as BMI and so on. Will these factors be adjusted in the primary models? Please specify. If so, will the same size be enough to make the model estimable?

5. Will analysis be conducted within each disease group? If not, will there be heterogeneity across disease groups?

Reviewer #2: ABSTRACT:

*There is no an exact discussion, pls improve.

Background:

the last sentence of the first paragraph:....correct inter-idividual to inter-individual

DISCUSSION:

Relatively moderate sample size is not a limitation. Remove this sentence. Actually your achieved power is %83 and %90 for two statistical analysis, and both power rates are in clinically acceptable limits. Below you will find your protocols of power analysis calculated by G-Power. You’d better write in a sentence the power and the calculated sample size in the methods section, and remove the limitation mentioned in the discussion section. Note: You can state small sample size as a limitation if the power is <%80.

Protocol of Power Analysis:

F tests - ANOVA: Fixed effects, omnibus, one-way (for group comparisons of the results of three groups: RA, osteoarthritis and Sarcopenia)

Analysis: A priori: Compute required sample size

Input: Effect size f = 0.4 (large, Cohen’s)

α err prob = 0.05

Power (1-β err prob) = 0.83

Number of groups = 3

Total sample size = 69

Actual power = 0.8365988

Protocol of Power Analysis:

4] -- Tuesday, May 12, 2026 -- 23:11:40

t tests - Means: Difference between two independent means (two groups) (for comparing the results of endurance exercise and muscle strength exercise groups)

Analysis: Post hoc: Compute achieved power

Input: Tail(s) = Two

Effect size d = 0.8 (large, Cohen’s)

α err prob = 0.05

Sample size group 1 = 35

Sample size group 2 = 34

Power (1-β err prob) = 0.9056359

Reviewer #3: The abstract requires one clear aim sentence, so does the introduction section.

Please refrain from AI-generated phrases.

Please pay attention to the use of tenses all throughout the text.

Each software and device should be given along with manufacturer and country of origin.

Does each keyword exist in MeSH browser?

Exercise-related benefits include increased mitochondrial biogenesis/enzyme activity, reconditioning of immune/inflammatory pathways, decreased endoplasmic reticulum stress. Please cite relevant papers (PMID: 33793075) and provide a deeper insight to the topic for readers.

The age cut-off should be explained.

RA and OA are rheumatic diseases, Sarcopenia can be observed is a distinct entity which can also accompany these diseases. The methodology is questionable in this context.

"We aim to include 69 participants" How could you end up with this sample size?

Future insights could be added.

The reference list could be updated and diversified.

DXA is the preferred abbreviation for Dual-energy X-ray absorptiometry

Does each author meet the first two criteria of ICMJE?

**********

what does this mean?). If published, this will include your full peer review and any attached files.

If you choose “no”, your identity will remain anonymous but your review may still be made public.

**Do you want your identity to be public for this peer review?**  For information about this choice, including consent withdrawal, please see our Privacy Policy

Reviewer #1: No

Reviewer #2: No

Reviewer #3: No

**********

[NOTE: If reviewer comments were submitted as an attachment file, they will be attached to this email and accessible via the submission site. Please log into your account, locate the manuscript record, and check for the action link "View Attachments". If this link does not appear, there are no attachment files.]

To ensure your figures meet our technical requirements, please review our figure guidelines: https://journals.plos.org/plosone/s/figures

You may also use PLOS’s free figure tool, NAAS, to help you prepare publication quality figures: https://journals.plos.org/plosone/s/figures#loc-tools-for-figure-preparation.

NAAS will assess whether your figures meet our technical requirements by comparing each figure against our figure specifications.

# Author comment: Author response to Decision Letter 1

**DOI:** 10.1371/journal.pone.0356181.r002

23 Jul 2026

Response to Reviewers’ Comments

Manuscript title: Effects of exercise on muscle strength and characteristics in rheumatic diseases and sarcopenia: Protocol for the Care for Muscle (C4M) Study

Response to Reviewers’ Comments

Manuscript title: Effects of exercise on muscle strength and characteristics in rheumatic diseases and sarcopenia: Protocol for the Care for Muscle (C4M) Study

Dear Dr. Akaras,

We sincerely thank the editor and the reviewers for their careful assessment of our manuscript and for their valuable comments and suggestions. Their feedback has helped us to improve both the clarity and the scientific quality of the manuscript. We have addressed each comment in detail below and revised the manuscript accordingly. All changes are indicated in the responses and in the revised manuscript.

Response to Academic Editor’s Comments

Comment 1. Manuscript formatting and PLOS ONE style requirements

Response: We have revised the manuscript according to the PLOS ONE formatting requirements and ensured that the file naming conventions and formatting guidelines have been followed.

Comment 2. Funding statement

Response and author action: We have removed all funding-related text from the manuscript, including the Acknowledgments section, as requested. The Funding Statement provided in the online submission system is correct and does not require any amendments. The updated Funding Statement is also included in the cover letter.

Comment 3. Ethics statement

Response and author action: We have removed the ethics statement from all sections except the Methods section, where it is now appropriately reported.

Comment 4. Figure 1 caption

Response and author action: We have added a caption for figure 1 as requested

5. If the reviewer comments include a recommendation to cite specific previously published works, please review and evaluate these publications to determine whether they are relevant and should be cited. There is no requirement to cite these works unless the editor has indicated otherwise.

Response to Reviewers’ Comments

We sincerely thank the reviewers for their careful evaluation of our manuscript and for their constructive comments and suggestions. We have addressed each comment below and revised the manuscript accordingly.

Reviewer #1: The authors intend to explore the association between muscle characteristics and exercise outcomes in RA, OA, and sarcopenia through an exploratory trial with 69 patients allocated to two arms (endurance and muscle strength exercises).

1. Line 35. The authors claimed 69 participants to be sufficient to capture variability in muscle characteristics. Is there any evidence or reasoning to support this argument? Considering 3 disease groups and 2 intervention groups, each analytical group will be only around 11 participants!

Response: Thank you for this important comment. We agree that the original wording suggested a level of statistical justification that was not adequately supported. The study is exploratory and was not powered for disease-specific subgroup analyses or interaction effects.

We agree that further clarification of the sample size rationale is warranted. The sample size of 69 participants was primarily chosen to ensure representation of the three clinical populations (RA, OA and sarcopenia) while maintaining feasibility for the extensive mechanistic assessments, including muscle biopsies and high-resolution respirometry. The study was designed as an exploratory mechanistic trial and the target sample size was determined pragmatically, balancing the feasibility of the extensive mechanistic assessments with the need to obtain sufficiently precise estimates of variability and intervention effects for future confirmatory studies.

As no studies have compared strength and endurance exercise in patients with RA, OA or sarcopenia, no reliable disease-specific between-group effect size was available to perform a formal a priori sample size calculation for the final study design. A convenient and feasible sample of a total of 69 patients will be included, taking into account a 15% dropout rate, 60 patients will be analysed of which 30 will be randomized to the strength group and 30 to the endurance group, stratified by diagnosis (OA, SARC and RA) and gender.

To provide additional context, we performed a post hoc evaluation using effect sizes reported in meta-analyses in healthy adults (Lopez P et al, Med. Sci. Sports Exerc 2021), which provided supportive context regarding the feasibility of the selected sample size for the primary comparison between exercise modalities. We have clarified this rationale in the Methods.

Author action: We have revised the sample size section accordingly and now explicitly state that the study is intended to generate exploratory estimates and inform future adequately powered trials.

Adapted in the manuscript (Page 13, line 393-405): This study was designed as an exploratory mechanistic trial because no previous studies have compared strength and endurance exercise in patients with rheumatoid arthritis, osteoarthritis or sarcopenia. Consequently, no reliable disease-specific between-group effect size was available to perform a formal a priori sample size calculation for the primary comparison. The target sample size was therefore determined pragmatically, balancing the feasibility of the extensive mechanistic assessments (including muscle biopsies, mitochondrial respirometry and inflammatory biomarker analyses) with the aim of including as many participants as possible within the available resources and study period. A total of 69 patients will be included, taking into account a 15% dropout rate, 60 patients will be analysed of which 30 will be randomized to the strength group and 30 to the endurance group, stratified by diagnosis (OA, SARC and RA) and gender. This resulted in a target sample size of 69 participants (23 per disease group). The study is intended to estimate intervention effects, variability and mechanistic associations to inform the design of future adequately powered confirmatory studies. The study is not powered for separate confirmatory analyses within each disease group

2. Line 228. Are peak power and torque the same or interchangeable? In the method for the primary outcome, it’s unclear which one will be analyzed as the primary outcome.

Response: Thank you for identifying this inconsistency. Peak power and torque are distinct biomechanical parameters and should not be used interchangeably. Our intended primary outcome is isokinetic knee extension peak torque measured using the Biodex dynamometer and normalized for body weight (Nm/kg).

Author action: We have revised the abstract, methods, outcome description and statistical analysis sections to consistently refer to peak torque as the primary outcome throughout the manuscript.

In addition to changes in the method section, we specified the text of the measurement of mitochondrial function (Page 11, Line 307-312):

This protocol involves sequential addition of glutamate, malate, and pyruvate to assess complex I supported leak respiration, followed by ADP to stimulate coupled oxidative phosphorylation (OXPHOS) via complex I (NADH-linked), cytochrome c to test for outer mitochondrial membrane integrity, succinate to assess complex II supported respiration (Succinate-linked), FCCP to reveal total uncoupled electron transport system (ETS) capacity, rotenone to inhibit complex I, and finally antimycin A to inhibit complex III and reveal residual oxygen consumption.

3. Line 403. Interaction is also among their interest. Will 69 participants be enough? What minimal detectable effect size can be identified with the proposed sample size? Considering the small sample size, will the effect be estimable?

Response: We agree that the proposed sample size is not sufficient to robustly detect small-to-moderate interaction effects. The interaction analyses are exploratory and intended to investigate potential signals that may inform future confirmatory studies. This is consistent with the study design, as the selected sample size was intended to address the primary intervention comparison and exploratory mechanistic objectives rather than to detect subgroup-specific or interaction effects.

Author action: Accordingly, we have revised the statistical analysis section to clarify that interaction estimates will be interpreted cautiously, with emphasis on effect sizes and confidence intervals rather than formal hypothesis testing.

We specified the text for the analyses for primary and secondary outcome:

Adapted in the manuscript (page 15, line 448-469)

1. Primary outcome (isokinetic muscle strength):

The effect of exercise type (strength vs. muscle endurance exercise) on isokinetic muscle strength (pre- vs. post-intervention) will be assessed using linear mixed models. Models will include fixed effects for exercise group, time (pre/post), exercise group, disease type, and gender. The time × exercise group interaction will be included to estimate differential changes in muscle strength between intervention groups. Random effects will account for individual variability.

2. Secondary outcome (Associations between muscle characteristics and exercise response)

ii) To explore whether associations between muscle characteristics and exercise response differ by exercise modality, models will be extended by including exercise group and interaction terms (muscle characteristic × exercise group). Given the exploratory nature of the study and the limited sample size, these analyses are intended to identify potential signals rather than provide definitive evidence of effect modification. Interaction estimates will be interpreted cautiously, with emphasis on estimated effect sizes and 95% confidence intervals rather than statistical significance testing.

iii) Adjusting for covariates. All models will be adjusted for the minimization factors (gender and disease type) and prespecified covariates (age and BMI). Given the exploratory nature of the study and the available sample size, the number of covariates will be restricted to reduce the risk of model overfitting.

4. Minimization ensures balance on disease type and gender, but not other factors, such as BMI and so on. Will these factors be adjusted in the primary models? Please specify. If so, will the same size be enough to make the model estimable?

Response: Thank you for this suggestion. In addition to the minimization factors (disease type and gender), age and BMI will be included as prespecified covariates in the regression models because of their known associations with muscle characteristics and training response.

Given the exploratory nature of the study and limited sample size, the number of covariates will be restricted to avoid model overfitting.

Author action: We have clarified this approach in the statistical analysis

Adapted in the manuscript (page 15, line 467-469)

5. Will analysis be conducted within each disease group? If not, will there be heterogeneity across disease groups?

Response: The primary analyses will be conducted on the combined study population, with disease type included as a fixed effect in the models. The study is not powered for separate confirmatory analyses within each disease group.

Potential heterogeneity across disease groups will be explored through disease-specific descriptive analyses and exploratory interaction terms. Any disease-specific findings will be interpreted as hypothesis-generating.

Reviewer #2: ABSTRACT:

*There is no an exact discussion, pls improve.

Response: Thank you for this comment. We are not entirely sure how to interpret this remark, as the abstract already includes a Discussion section. We would appreciate further clarification regarding the specific aspects that the reviewer feels should be improved.

Background:

the last sentence of the first paragraph:....correct inter-idividual to inter-individual

Response and authors action: Thank you. The spelling error has been corrected (page 4, line 77).

DISCUSSION:

Relatively moderate sample size is not a limitation. Remove this sentence. Actually your achieved power is %83 and %90 for two statistical analysis, and both power rates are in clinically acceptable limits. Below you will find your protocols of power analysis calculated by G-Power. You’d better write in a sentence the power and the calculated sample size in the methods section, and remove the limitation mentioned in the discussion section. Note: You can state small sample size as a limitation if the power is <%80.

Protocol of Power Analysis:

F tests - ANOVA: Fixed effects, omnibus, one-way (for group comparisons of the results of three groups: RA, osteoarthritis and Sarcopenia)

Analysis: A priori: Compute required sample size

Input: Effect size f = 0.4 (large, Cohen’s)

α err prob = 0.05

Power (1-β err prob) = 0.83

Number of groups = 3

Total sample size = 69

Actual power = 0.8365988

Protocol of Power Analysis:

4] -- Tuesday, May 12, 2026 -- 23:11:40

t tests - Means: Difference between two independent means (two groups) (for comparing the results of endurance exercise and muscle strength exercise groups)

Analysis: Post hoc: Compute achieved power

Input: Tail(s) = Two

Effect size d = 0.8 (large, Cohen’s)

α err prob = 0.05

Sample size group 1 = 35

Sample size group 2 = 34

Power (1-β err prob) = 0.9056359

Response: We thank the reviewer for this helpful comment and for performing the G*Power calculations. These calculations were very helpful in performing our thoughts for determining the sample size. We agree that further justification of the selected sample size is warranted.

The proposed calculations are based on an assumed large intervention effect (Cohen's d = 0.8), for which no disease-specific evidence is currently available. No studies have compared strength and endurance exercise in patients with RA, OA or sarcopenia, and therefore no disease-specific between-group effect size was available to perform a formal a priori sample size calculation for the final study design.

This study was designed as an exploratory mechanistic trial rather than a confirmatory efficacy trial. Consequently, the target sample size of 69 participants was determined pragmatically, balancing the feasibility of the extensive mechanistic assessments (including muscle biopsies and mitochondrial analyses) with the aim of including as many participants as possible to obtain preliminary estimates of variability and effect sizes for future confirmatory studies. Hereby, taking into account a 15% dropout rate, 60 patients will be analysed of which 30 will be randomized to the strength group and 30 to the endurance group, stratified by diagnosis (OA, SARC and RA) and gender.

To place the selected sample size into context, we performed a post hoc evaluation using effect sizes reported in meta-analyses of resistance training in healthy adults, which consistently report small-to-moderate between-group effects (approximately Cohen's d = 0.3–0.4) for muscle strength outcomes (Lopez P et al, Med. Sci. Sports Exerc 2021). These findings provide reassurance that the planned sample size is likely to be adequate for the primary intervention comparison. However, this post hoc evaluation should be regarded as supportive rather than as an a priori justification of the sample size.

Author action: We agree that the wording of the limitation section could be improved. We have revised the discussion to clarify that the limitation relates not to detecting overall group differences, but rather to the ability to estimate subgroup-specific and interaction effects with precision. The study should therefore be regarded as exploratory and hypothesis-generating

Adapted in the manuscript (page 17, line 517-524)

A limitation of this trial is that the sample size, while adequate for the exploratory objectives of the study, limits the estimation of precision of subgroup-specific and interaction effects. The heterogeneity in disease pathology and baseline muscle characteristics further complicates the interpretation of disease-specific analyses. Consequently, multivariable and interaction analyses should be regarded as hypothesis-generating rather than confirmatory, with emphasis placed on effect si

Attachment Submitted filename: Response to Reviewers 16-07-26. docx.docx

# Peer review: Decision Letter 1

**DOI:** 10.1371/journal.pone.0356181.r003

**Authors:** Heitor Ribeiro

30 Jul 2026

<p>Effects of exercise on muscle strength and characteristics in rheumatic diseases and sarcopenia: Protocol for the Care for Muscle (C4M) Study

PONE-D-25-67495R1

Dear Dr. de Rooij,

We’re pleased to inform you that your manuscript has been judged scientifically suitable for publication and will be formally accepted for publication once it meets all outstanding technical requirements.

Within one week, you’ll receive an e-mail detailing the required amendments. When these have been addressed, you’ll receive a formal acceptance letter and your manuscript will be scheduled for publication.

An invoice will be generated when your article is formally accepted. Please note, if your institution has a publishing partnership with PLOS and your article meets the relevant criteria, all or part of your publication costs will be covered. Please make sure your user information is up-to-date by logging into Editorial Manager at Editorial Manager® and clicking the ‘Update My Information' link at the top of the page. For questions related to billing, please contact billing support.

If your institution or institutions have a press office, please notify them about your upcoming paper to help maximize its impact. If they’ll be preparing press materials, please inform our press team as soon as possible -- no later than 48 hours after receiving the formal acceptance. Your manuscript will remain under strict press embargo until 2 pm Eastern Time on the date of publication. For more information, please contact onepress@plos.org.

Kind regards,

Heitor S. Ribeiro, PhD

Academic Editor

PLOS One

Additional Editor Comments (optional):

The reviewers have no further comments and the study protocol is suitable for publication.

Reviewers' comments:

Reviewer's Responses to Questions

**Comments to the Author**

1. Does the manuscript provide a valid rationale for the proposed study, with clearly identified and justified research questions?

Reviewer #1: Yes

**********

2. Is the protocol technically sound and planned in a manner that will lead to a meaningful outcome and allow testing the stated hypotheses?

Reviewer #1: Yes

**********

3. Is the methodology feasible and described in sufficient detail to allow the work to be replicable?

Reviewer #1: Yes

**********

4. Have the authors described where all data underlying the findings will be made available when the study is complete?

The PLOS Data policy

Reviewer #1: No

**********

5. Is the manuscript presented in an intelligible fashion and written in standard English?

Reviewer #1: Yes

**********

Please use the space provided to explain your answers to the questions above and, if applicable, provide comments about issues authors must address before this protocol can be accepted for publication. You may also include additional comments for the author, including concerns about research or publication ethics.

You may also provide optional suggestions and comments to authors that they might find helpful in planning their study.

Reviewer #1: Thank you for the response. The authors have satisfactory addressed all the raised concerns. This reviewer doesn't have further concerns.

**********

what does this mean?). If published, this will include your full peer review and any attached files.

If you choose “no”, your identity will remain anonymous but your review may still be made public.

**Do you want your identity to be public for this peer review?**  For information about this choice, including consent withdrawal, please see our Privacy Policy

Reviewer #1: No

**********

# Editor report: Acceptance letter

**DOI:** 10.1371/journal.pone.0356181.r004

**Authors:** Heitor Ribeiro

PONE-D-25-67495R1

PLOS One

Dear Dr. de Rooij,

I'm pleased to inform you that your manuscript has been deemed suitable for publication in PLOS One. Congratulations! Your manuscript is now being handed over to our production team.

At this stage, our production department will prepare your paper for publication. This includes ensuring the following:

* All references, tables, and figures are properly cited

* All relevant supporting information is included in the manuscript submission,

* There are no issues that prevent the paper from being properly typeset

You will receive further instructions from the production team, including instructions on how to review your proof when it is ready. Please keep in mind that we are working through a large volume of accepted articles, so please give us a few days to review your paper and let you know the next and final steps.

Lastly, if your institution or institutions have a press office, please let them know about your upcoming paper now to help maximize its impact. If they'll be preparing press materials, please inform our press team within the next 48 hours. Your manuscript will remain under strict press embargo until 2 pm Eastern Time on the date of publication. For more information, please contact onepress@plos.org.

You will receive an invoice from PLOS for your publication fee after your manuscript has reached the completed accept phase. If you receive an email requesting payment before acceptance or for any other service, this may be a phishing scheme. Learn how to identify phishing emails and protect your accounts at https://explore.plos.org/phishing.

If we can help with anything else, please email us at customercare@plos.org.

Thank you for submitting your work to PLOS One and supporting open access.

Kind regards,

PLOS One Editorial Office Staff

on behalf of

Dr. Heitor S. Ribeiro

Academic Editor

PLOS One
